# Supplementary material for: The interactome of intact mitochondria by cross-linking mass spectrometry provides evidence for coexisting respiratory supercomplexes
Source: Mol Cell Proteomics. 2017 Dec 8;17(2):216–32. doi: 10.1074/mcp.RA117.000470 (PMC5795388; doi:10.1074/mcp.RA117.000470)
Supplement: Supplemental Data [file supp_RA117.000470_133922_0_supp_23978_fzffwf.zip › spectra_annotation/mito_DR_spectra_annotation/407-2-4-1-7-1.pdf]

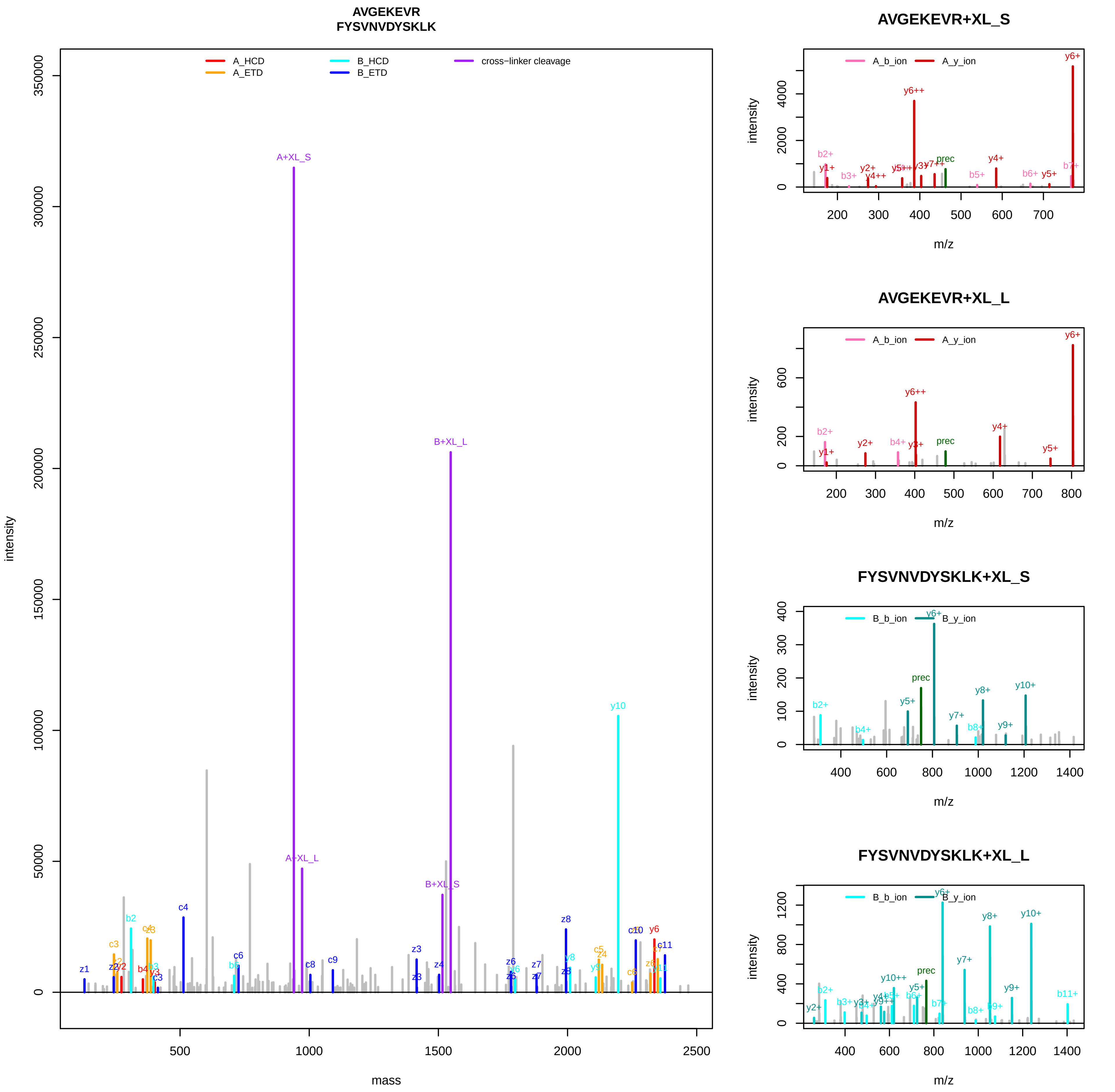
AVGEKEVR
FYSVNVDYSKLK
A_HCD
A_ETD
B_HCD
B_ETD
cross−linker cleavage
A+XL_S
B+XL_L
A+XL_L
B+XL_S
y10
intensity
mass
0
50000
100000
150000
200000
250000
300000
350000
500
1000
1500
2000
2500
AVGEKEVR+XL_S
A_b_ion
A_y_ion
y6+
y6++
prec
y4+
b2+
y1+
b3+
y2+
b5+
b6+
y5+
b7+
m/z
AVGEKEVR+XL_L
y6+
y6++
y4+
prec
y2+
b4+
y5+
b2+
y1+
FYSVNVDYSKLK+XL_S
B_b_ion
B_y_ion
y6+
prec
y5+
y8+
y10+
y7+
y9+
b2+
b4+
b8+
FYSVNVDYSKLK+XL_L
y6+
y8+
y10+
y7+
prec
y9+
b2+
b11+
b7+
b8+
b9+
